# Vortioxetine-Associated Rapid Eye Movement (REM) Sleep Alterations: A Focused Narrative Review of Human Polysomnographic Evidence

**DOI:** 10.7759/cureus.116174

**Published:** 2026-09-13

**Authors:** Binh Pham, Sakeena Siddiq

**Affiliations:** 1 Sleep Medicine, Southwest Healthcare Medical Education Consortium, Temecula, USA; 2 Psychiatry, Western University of Health Sciences, Pomona, USA

**Keywords:** 5-ht receptors, clinical pharmacology, polysomnography (psg), rem (rapid eye movement), rem sleep regulation, sert, sleep latency, sleep pharmacology, sleep-wake cycles, vortioxetine

## Abstract

This focused narrative review examines the relationship between vortioxetine and rapid eye movement (REM) sleep and considers the potential clinical significance of REM-related changes observed during treatment. Direct human polysomnographic evidence remains very limited and is derived principally from two studies. The review integrates relevant sleep neurobiology, vortioxetine pharmacology, and human polysomnographic findings while distinguishing biological plausibility from demonstrated clinical significance. The available evidence is interpreted cautiously because changes in individual REM measures do not necessarily represent a uniform alteration of REM physiology and an objective polysomnographic effect should not be assumed to reflect a therapeutic mechanism. At present, vortioxetine-associated REM changes are best regarded as a pharmacodynamic effect whose persistence, dose dependence, underlying mechanism, and clinical relevance remain uncertain. These changes have not been established as surrogate markers of antidepressant response or other meaningful patient outcomes. Further prospective research integrating objective sleep measures with symptoms, subjective sleep quality, daytime functioning, cognition, and longer-term outcomes is needed to clarify the significance of vortioxetine-associated REM alterations.

## Introduction and background

Normal sleep is a highly organized neurophysiologic process composed of recurring cycles of non-rapid eye movement (NREM) and rapid eye movement (REM) sleep that alternate throughout the night. NREM sleep consists of stages N1, N2, and N3, which are distinguished by characteristic electroencephalographic features and increasing depth of NREM sleep. In contrast, REM sleep is characterized by cortical activation, rapid eye movements, skeletal muscle atonia, and autonomic variability. These stages represent distinct but interacting neurophysiologic states regulated by distributed sleep-wake networks [1,2].

REM sleep is actively generated and maintained through coordinated interactions among neuronal populations distributed throughout the brainstem, hypothalamus, and forebrain. Experimental models support a distributed network involving glutamatergic, gamma-aminobutyric acid (GABA)-mediated, cholinergic, serotonergic, and noradrenergic signaling [3]. Serotonergic neurons within the dorsal raphe nucleus and noradrenergic neurons within the locus coeruleus exhibit pronounced state-dependent changes in activity, with reduced firing during NREM sleep and minimal or near-absent activity during REM sleep [4,5]. These state-dependent changes provide a neurobiological framework for understanding how pharmacologic modulation of monoaminergic neurotransmission may alter REM sleep architecture.

Alterations in REM sleep are among the most frequently described polysomnographic findings in major depressive disorder. Reported abnormalities include shortened REM latency, increased REM density, greater REM expression during the early portion of the sleep period, and accompanying disturbances in sleep continuity, although the magnitude and consistency of these findings vary across individuals and study populations [6,7]. Importantly, REM latency, REM duration, REM percentage, REM density, and REM fragmentation represent related but distinct polysomnographic measures and should not be considered interchangeable indicators of REM physiology. Accordingly, treatment-related changes should be described according to the specific REM parameter affected rather than generalized as uniform suppression of REM physiology. Although these abnormalities have contributed to longstanding interest in REM dysregulation as a feature of depression, they are not sufficiently specific to function as standalone diagnostic biomarkers. Accordingly, changes in individual REM parameters during antidepressant treatment should be interpreted within the broader context of depression-associated sleep physiology rather than assumed to represent the normalization of a single underlying abnormality.

Recognition of REM abnormalities in depression has prompted extensive investigation into the effects of antidepressant treatment on REM sleep architecture. Human polysomnographic studies of serotonergic antidepressants have demonstrated the prolongation of REM latency and reductions in REM sleep expression, although the magnitude and pattern of these effects vary among individual medications [8,9]. These findings are consistent with pharmacologic modulation of neurotransmitter systems involved in REM regulation but do not establish that treatment-induced REM alterations mediate antidepressant efficacy. Accordingly, objective changes in REM sleep architecture should be distinguished from improvements in depressive symptoms, subjective sleep quality, or other clinically meaningful outcomes.

Vortioxetine is a multimodal serotonergic antidepressant approved for the treatment of major depressive disorder. Its pharmacologic profile includes the inhibition of the serotonin transporter (SERT) and modulation of multiple serotonin, or 5-hydroxytryptamine (5-HT), receptor subtypes, including 5-HT1A receptor agonism, 5-HT1B receptor partial agonism, and 5-HT3 and 5-HT7 receptor antagonism [10]. These simultaneous actions provide biological plausibility for effects on sleep-wake regulation but do not establish which individual pharmacologic target, if any, accounts for changes observed in human sleep architecture. Direct human polysomnographic evidence remains sparse and is derived principally from two studies: a controlled study in healthy men and a clinical study in adolescents with major depressive disorder [11,12]. The small size of this evidence base, together with substantial differences in population, study design, dosing, and treatment duration, limits the strength and generalizability of inferences regarding vortioxetine-associated changes in REM sleep. These differences are therefore important when weighing the subsequent mechanistic discussion and clinical interpretation.

The purpose of this focused narrative review is to critically evaluate the available human polysomnographic evidence regarding vortioxetine-associated alterations in REM sleep architecture, with particular attention to REM latency and measures of REM sleep expression. The review also examines the neurobiological regulation of REM sleep and the pharmacologic properties of vortioxetine that provide biological plausibility for the observed sleep effects while distinguishing these mechanistic considerations from demonstrated human polysomnographic findings. Particular emphasis is placed on differences in study design, population, dosing, and treatment duration to clarify what can and cannot presently be inferred regarding the clinical significance of vortioxetine-associated REM sleep changes. A summary of normal sleep architecture, its principal neurobiological features, and the reported effects of serotonergic antidepressants on individual sleep stages is provided in Table 1.

**Table 1:** Normal sleep architecture, principal neurobiological features, and reported effects of serotonergic antidepressants This table summarizes the principal characteristics of normal sleep stages, their predominant neurobiological mechanisms, and the reported effects of serotonergic antidepressants on sleep architecture. The reported pharmacologic effects are qualitative and synthesized from the cited literature. Individual findings may vary according to the specific antidepressant, receptor-binding profile, dosage, treatment duration, underlying neuropsychiatric disorder, concomitant therapies, and polysomnographic study methodology. GABA: gamma-aminobutyric acid; NREM: non-rapid eye movement; REM: rapid eye movement

Sleep stage	Principal physiologic features	Principal neurobiological features	Reported effects of serotonergic antidepressants
N1 sleep	Transitional stage between wakefulness and sleep, characterized by the attenuation of alpha activity and emergence of low-amplitude mixed-frequency electroencephalographic activity [1,2]	Progressive reduction in wake-promoting activity with increasing influence of sleep-promoting networks [2]	Effects are variable; some serotonergic antidepressants may increase lighter sleep or alter transitions between wakefulness and sleep [9]
N2 sleep	Characterized by sleep spindles and K-complexes and normally constitutes a substantial proportion of total sleep time [1,2]	Thalamocortical oscillatory activity contributes to the stabilization and maintenance of the sleep state [2]	Effects are variable across medications and studies, with no uniform change consistently demonstrated [9]
N3 sleep	Deep NREM sleep characterized by high-amplitude, low-frequency slow waves and an increased arousal threshold [1,2]	Prominent cortical and thalamocortical synchronization influenced by homeostatic sleep pressure and reduced activity of wake-promoting systems [2]	Effects are heterogeneous and medication-dependent; consistent enhancement or suppression of N3 sleep has not been demonstrated across serotonergic antidepressants [9]
REM sleep	Characterized by cortical activation, rapid eye movements, skeletal muscle atonia, and autonomic variability [1,2]	Generated by distributed REM-promoting and REM-suppressing networks involving glutamatergic, GABA-mediated, cholinergic, serotonergic, and noradrenergic signaling [3]	Serotonergic antidepressants commonly prolong REM latency and may reduce REM duration or percentage, although the magnitude and pattern vary among individual medications [8,9]

## Review

Literature search strategy

A focused narrative review of the literature was conducted to identify human polysomnographic studies evaluating the effects of vortioxetine on sleep architecture and to provide the neurobiological and pharmacologic context necessary for the interpretation of these findings. PubMed was searched in August 2026 without restriction on publication date, and searches were limited to English-language publications. Google Scholar was used as a supplementary discovery and citation-tracking tool rather than as an exhaustive bibliographic database. Reference lists of relevant primary studies and review articles were also manually examined to identify additional publications.

For PubMed, the principal search for direct human polysomnographic evidence used the following search string: ("vortioxetine" OR "Lu AA21004") AND ("sleep" OR "sleep architecture" OR "polysomnography" OR "rapid eye movement sleep" OR "REM sleep" OR "REM latency" OR "REM duration" OR "REM percentage"). No field restrictions were applied. Supporting pharmacologic searches used ("vortioxetine" OR "Lu AA21004") AND ("serotonin transporter" OR "SERT" OR "5-HT1A" OR "5-HT1B" OR "5-HT1D" OR "5-HT3" OR "5-HT7"). Additional targeted searches addressing the serotonergic regulation of REM sleep and the effects of serotonergic antidepressants on sleep architecture were used to provide broader neurobiological and clinical context.

Google Scholar was searched using targeted combinations of the same concepts, including "vortioxetine" polysomnography REM, "vortioxetine" "REM sleep", "vortioxetine" "REM latency", "Lu AA21004" sleep REM, and combinations of "vortioxetine" with relevant serotonergic pharmacologic terms. Because Google Scholar dynamically ranks search results, it was not treated as an exhaustive database search. The exact number of Google Scholar results screened was not prospectively recorded, and no predefined stopping rule was applied. Its role was therefore limited to identifying potentially relevant publications and supporting citation tracking.

For the direct human evidence, publications were considered if they involved human participants exposed to vortioxetine, objective polysomnographic assessment, and reporting of treatment-related REM sleep or other sleep-architecture outcomes. Principal direct evidence was restricted to studies designed to evaluate vortioxetine-associated sleep architecture in participant samples rather than individual case reports dominated by major comorbid sleep disorders. Such case reports were considered separately as contextual evidence when relevant. Publications without objective polysomnographic assessment or without treatment-related sleep-architecture outcomes were not classified as principal direct evidence.

Preclinical, pharmacologic, neurophysiologic, and broader antidepressant sleep studies were considered separately when they addressed predefined areas relevant to the interpretation of the human findings: the neurobiological regulation of REM sleep, the established pharmacologic actions of vortioxetine, or the effects of serotonergic antidepressants on REM-related measures. Primary experimental and clinical studies were preferentially used for specific mechanistic or pharmacologic claims, whereas authoritative reviews were used primarily for broader synthesis and background. An evidentiary hierarchy was applied in which direct human polysomnographic evidence was given the greatest weight for characterizing vortioxetine-associated sleep effects, human pharmacologic and neurophysiologic evidence was used for interpretation where applicable, and preclinical studies were used to provide biological plausibility rather than establish mechanisms in humans.

Titles and abstracts were reviewed for relevance, followed by full-text assessment of potentially eligible human publications. Seven human publications with potentially relevant sleep-related outcomes were assessed for direct evidence, and two studies met the criteria for principal direct human polysomnographic evidence [11,12]. The remaining publications were not classified as principal direct evidence because they consisted of individual case reports with major comorbid sleep disorders, did not report treatment-related sleep-architecture outcomes, evaluated physiologic measures other than conventional sleep architecture, or relied on subjective sleep assessment without polysomnography. For the two principal studies, extracted information included study population, sample size, clinical indication, vortioxetine dose, treatment duration, study design, comparator or control condition, polysomnographic methodology, REM-related outcomes, other reported sleep-architecture findings, and major study limitations.

The methodological characteristics of the two principal studies were evaluated qualitatively with attention to design-specific sources of bias. For the controlled crossover study, considerations included the small sample, short treatment exposure, and potential period or carryover effects, although the washout interval between treatment periods reduced the likelihood of substantial carryover [11]. For the uncontrolled before-and-after study, important considerations included the absence of a control group, potential confounding by changes in depressive illness, prior antidepressant exposure, time-related effects, and possible regression to the mean [12]. These design-specific limitations were incorporated into the relative weighting and interpretation of the two studies. No formal risk-of-bias instrument was applied.

Because the direct human polysomnographic evidence was limited and the two principal studies differed substantially in population, study design, dosing, treatment duration, and measured sleep outcomes, quantitative pooling was not considered appropriate. The findings were therefore synthesized qualitatively. This article was designed as a focused narrative review rather than a systematic review or meta-analysis. The restriction to English-language publications, reliance primarily on PubMed, and supplementary use of dynamically ranked Google Scholar results may have introduced language, database-selection, and discretionary-selection bias and may have resulted in the omission of relevant evidence. These limitations are addressed further in the "Limitations and Future Directions" section.

Neurobiological regulation of sleep and REM sleep

Sleep is an actively regulated neurophysiologic process governed in part by the interaction between homeostatic sleep pressure and circadian timing, commonly conceptualized as Process S and Process C, respectively [2,13]. These processes influence the timing and propensity for sleep, whereas transitions among wakefulness, NREM sleep, and REM sleep arise from coordinated activity across distributed neuronal networks involving the hypothalamus, brainstem, basal forebrain, thalamus, and cerebral cortex [2]. Sleep architecture therefore reflects the dynamic interaction of homeostatic, circadian, and neurochemical mechanisms rather than a passive progression through behavioral states.

Transitions between sleep and wakefulness are regulated through reciprocal interactions between sleep-promoting and wake-promoting neuronal systems. GABAergic neurons within the preoptic hypothalamus inhibit major components of the ascending arousal system, including histaminergic, noradrenergic, serotonergic, and cholinergic populations, whereas these arousal systems reciprocally suppress sleep-promoting activity during wakefulness [2,14]. This reciprocal organization has been conceptualized as a flip-flop switching mechanism that favors relatively rapid and stable transitions between behavioral states. Orexin-producing neurons of the lateral hypothalamus provide additional excitatory input to wake-promoting systems and contribute to the stabilization of sleep-wake state transitions [14].

Building on the neurobiological framework outlined above, REM sleep emerges from coordinated interactions among REM-promoting and REM-suppressing circuits rather than from a single anatomic center. Experimental models implicate glutamatergic, GABA-mediated, cholinergic, serotonergic, and noradrenergic signaling in the initiation, maintenance, and termination of REM sleep [3,15]. The balance among these systems contributes to characteristic REM features, including cortical activation, rapid eye movements, and skeletal muscle atonia. This network organization provides a physiologic basis for understanding why medications that modify monoaminergic neurotransmission may alter specific REM-related polysomnographic measures.

Pharmacologic alteration of serotonergic neurotransmission can therefore modify individual polysomnographic measures of REM sleep, including REM latency and the amount of REM sleep expressed during the sleep period [8,9]. However, these measures reflect distinct aspects of REM physiology, and changes in one parameter should not be assumed to represent uniform suppression of the REM state. Moreover, a medication-associated alteration in REM architecture constitutes a pharmacodynamic observation and does not by itself demonstrate therapeutic benefit, normalization of depression-associated sleep physiology, or mediation of antidepressant efficacy. This distinction is particularly important when interpreting the effects of a multimodal serotonergic agent such as vortioxetine.

Serotonergic regulation of REM sleep

Serotonergic neurotransmission is an important component of REM sleep regulation and one of the principal monoaminergic systems involved in transitions between behavioral states. Serotonergic neurons located primarily within the dorsal raphe nucleus exhibit state-dependent firing patterns, with greater activity during wakefulness, reduced activity during NREM sleep, and minimal or near-absent activity during REM sleep [4]. These changes occur alongside reductions in noradrenergic activity within the locus coeruleus and within a broader reciprocal network involving cholinergic, glutamatergic, and GABAergic signaling [3-5]. Interactions among these systems contribute to the initiation and maintenance of REM sleep and to characteristic features such as cortical activation, rapid eye movements, and skeletal muscle atonia.

Because serotonergic activity normally changes across the sleep-wake cycle, pharmacologic enhancement of serotonergic neurotransmission can modify REM sleep architecture. Human polysomnographic studies of serotonergic antidepressants have demonstrated the prolongation of REM latency and reductions in measures of REM sleep expression, although the magnitude and pattern of these effects vary among medications and across study conditions [8,9]. These findings support serotonergic modulation as an important component of antidepressant-related REM changes but do not establish a uniform effect across all REM-related measures or a single serotonergic mechanism shared by all antidepressants. This framework provides the neurobiological context for considering how the multimodal pharmacology of vortioxetine may influence REM-related sleep measures.

Pharmacology of vortioxetine relevant to REM sleep

Vortioxetine is a multimodal serotonergic antidepressant whose pharmacologic profile combines the inhibition of the SERT with direct actions at several serotonin, or 5-HT, receptor subtypes. It acts as a 5-HT1A receptor agonist, a 5-HT1B receptor partial agonist, and an antagonist at 5-HT1D, 5-HT3, and 5-HT7 receptors [10,16-18]. This combination of transporter inhibition and receptor modulation distinguishes vortioxetine pharmacologically from antidepressants whose serotonergic actions are mediated predominantly through SERT inhibition [10,16]. Because these targets differ in affinity, anatomical distribution, and downstream signaling, the pharmacologic effects of vortioxetine are better conceptualized as the integrated consequence of multiple serotonergic actions than as the effect of any single receptor interaction.

SERT inhibition provides the most direct link between vortioxetine and the established effects of enhanced serotonergic neurotransmission on REM sleep. Inhibition of serotonin reuptake increases extracellular serotonin and prolongs serotonergic signaling within neural circuits involved in sleep-state regulation [10,16]. Given the state-dependent reduction in serotonergic neuronal activity that normally accompanies REM sleep, enhanced serotonergic transmission provides a biologically plausible basis for the prolongation of REM latency and reductions in REM sleep expression. In healthy volunteers, the relationship between estimated SERT occupancy and REM-related polysomnographic changes differed between vortioxetine and paroxetine despite comparable levels of transporter occupancy [11]. This finding suggests that SERT occupancy alone may not fully explain the observed differences in REM-related effects. However, the study does not establish the basis for this divergence, which could reflect differences in receptor-mediated pharmacology, drug exposure, pharmacokinetics, downstream network effects, or other drug-specific factors.

Among the receptor-mediated actions of vortioxetine, 5-HT3 receptor antagonism has received the most direct preclinical experimental attention in relation to sleep, although no human study has isolated its contribution to vortioxetine-associated REM changes. In rodents, both vortioxetine and paroxetine acutely delayed REM onset and reduced REM episodes, whereas their effects differed during repeated administration [17]. Pharmacologic manipulation of the 5-HT3 receptor further modified these REM-related effects, leading the investigators to propose that 5-HT3 receptor antagonism may contribute to the differentiated sleep effects of vortioxetine [17]. Separate preclinical experiments demonstrated that vortioxetine-mediated 5-HT3 receptor blockade can influence glutamatergic and serotonergic neurotransmission through effects involving GABAergic interneurons [18]. These findings provide biological plausibility for a potential contribution of 5-HT3 antagonism to vortioxetine-associated REM changes, but the evidence remains preclinical and does not establish that this receptor accounts for the effects observed in humans.

The additional receptor actions of vortioxetine, including 5-HT1A receptor agonism, 5-HT1B receptor partial agonism, and 5-HT1D and 5-HT7 receptor antagonism, may further modify serotonergic signaling and interactions with other neurotransmitter systems [10,16,18]. Their individual contributions to human REM sleep, however, have not been isolated experimentally. Accordingly, the human polysomnographic changes associated with vortioxetine should be interpreted as a whole-drug pharmacodynamic effect rather than assigned to SERT inhibition or any individual receptor action. Pharmacologic and preclinical studies provide biological plausibility for potential mechanisms, whereas determination of the actual sleep-architecture effects requires direct human polysomnographic evidence. The established pharmacologic actions of vortioxetine and their proposed relevance to REM sleep are summarized in Table 2.

**Table 2:** Pharmacologic actions of vortioxetine and evidence relevant to REM sleep Established pharmacologic actions are distinguished from human sleep evidence, preclinical findings, and mechanistic interpretation to make differences in evidentiary strength explicit. Human polysomnographic studies evaluate vortioxetine as a complete drug and do not isolate the contribution of individual receptor actions. Preclinical findings, particularly those involving 5-HT3 receptor antagonism, provide biological plausibility but should not be interpreted as evidence of receptor-specific causality in humans. 5-HT: 5-hydroxytryptamine; GABA: gamma-aminobutyric acid; PSG: polysomnography; REM: rapid eye movement; SERT: serotonin transporter

Pharmacologic target	Established pharmacologic action	Human sleep evidence	Preclinical sleep/mechanistic evidence	Interpretation
SERT	Inhibits serotonin reuptake and increases serotonergic signaling [10,16]	Human PSG demonstrates REM-related changes during vortioxetine exposure. The relationship between estimated SERT occupancy and REM effects differed from paroxetine [11]	Established serotonergic modulation provides biological plausibility for effects on REM regulation	Provides the most direct pharmacologic link to serotonergic effects on REM sleep, but SERT occupancy alone does not fully explain between-drug differences and does not establish the mechanism of the observed REM changes
5-HT1A	Agonist [10,16]	No human study has isolated the contribution of 5-HT1A activity to vortioxetine-associated REM changes	May modify serotonergic signaling through pre- and postsynaptic mechanisms	Mechanistically plausible, but receptor-specific effects on human REM sleep remain unestablished
5-HT1B	Partial agonist [10,16]	No receptor-specific human sleep evidence	May influence serotonergic and downstream neurotransmitter release	Potential contribution remains inferential
5-HT1D	Antagonist [17,18]	No receptor-specific human sleep evidence	Pharmacologic action may contribute to the integrated serotonergic effects of vortioxetine	Role in human REM regulation remains uncertain
5-HT3	Antagonist [10,16-18]	No human study has isolated the contribution of 5-HT3 antagonism to vortioxetine-associated REM changes	Rodent studies show the modification of REM-related effects and effects on serotonergic, glutamatergic, and GABAergic signaling [17,18]	Has the strongest sleep-specific preclinical support among the receptor-mediated actions, but remains a mechanistic hypothesis rather than an established human mechanism
5-HT7	Antagonist [10,16]	No receptor-specific human sleep evidence	Potential relevance to sleep-wake regulation based on serotonergic pharmacology	Biologically plausible, but its contribution to human REM changes has not been demonstrated
Integrated vortioxetine pharmacology	Combined SERT inhibition and modulation of multiple 5-HT receptor subtypes [10,16]	Human PSG reflects the effect of the complete drug rather than isolated receptor actions [11,12]	Preclinical findings provide potential explanations for how multiple actions may interact [17,18]	Human REM-related changes should be interpreted as whole-drug pharmacodynamic effects; current evidence cannot assign them to an individual pharmacologic target

Human polysomnographic evidence

Direct human polysomnographic evidence evaluating the effects of vortioxetine on REM sleep remains sparse and is derived principally from two studies with substantially different populations and experimental designs: a controlled crossover pharmacodynamic study in healthy adult men and an uncontrolled before-and-after clinical study in adolescents with major depressive disorder [11,12]. These designs address complementary but distinct questions. Controlled investigation in healthy volunteers provides stronger evidence for a direct medication-related effect on sleep physiology because it is less influenced by depressive illness or antidepressant response, whereas investigation in patients with major depressive disorder provides greater clinical relevance but introduces potential contributions from the underlying disorder and changes in clinical state during treatment. Interpretation also requires attention to the specific polysomnographic outcomes assessed. REM latency, total REM duration, REM percentage, REM density, and REM fragmentation represent related but distinct dimensions of REM physiology and should not be treated as interchangeable measures of a single phenomenon.

Wilson et al. conducted a randomized, double-blind, placebo-controlled, active-comparator, four-way crossover study in 24 healthy young men, of whom 19 completed all treatment periods [11]. Participants received vortioxetine 20 mg/day, vortioxetine 40 mg/day, paroxetine 20 mg/day, and placebo for three consecutive days during separate treatment periods, with at least three weeks between exposures. The short-term vortioxetine doses were used as loading regimens intended to approximate steady-state exposure associated with clinically used daily doses of 10 and 20 mg, respectively. Compared with placebo, both vortioxetine conditions significantly prolonged REM onset latency and reduced total REM sleep. On the third treatment night, the mean REM onset latency was 127 ± 45 minutes with vortioxetine 20 mg and 231 ± 65 minutes with vortioxetine 40 mg, compared with 63 ± 18 minutes with placebo; corresponding total REM sleep times were 63 ± 20, 41 ± 22, and 108 ± 21 minutes, respectively [11]. The mean total sleep time was 403 ± 44 minutes with vortioxetine 20 mg and 393 ± 51 minutes with vortioxetine 40 mg, compared with 413 ± 29 minutes with placebo, with a significant reduction in total sleep time reported for the 40-mg condition [11]. The larger REM-related changes observed with the 40-mg loading condition therefore represent a descriptive exposure-related gradient rather than a formally demonstrated dose-response relationship. Interpretation of the reduction in absolute REM duration at 40 mg also requires caution because the concurrent reduction in total sleep time may have contributed to the lower amount of REM sleep obtained. Accordingly, these findings should not be taken as evidence of either a selective REM-specific effect or a clinically reproducible dose-response relationship without accounting for the broader changes in sleep architecture. Vortioxetine also increased stage N1 sleep, and the 40-mg condition increased wake after sleep onset, further indicating that its acute effects were not confined exclusively to REM sleep.

The pharmacokinetic-pharmacodynamic analysis in the Wilson et al. study provides a related but distinct observation. Although vortioxetine and paroxetine produced comparable estimated SERT occupancy at the studied exposures, the relationship between SERT occupancy and REM-related changes differed between the two medications [11]. This finding suggests that SERT inhibition alone may not fully account for the REM-related effects observed with vortioxetine. However, it does not identify which additional pharmacologic action contributes to the difference and cannot establish a receptor-specific mechanism. The crossover design therefore provides relatively strong evidence that short-term vortioxetine exposure can directly alter REM physiology in humans, independent of changes in depressive illness. Its interpretation remains limited by the small sample, exclusively male healthy-volunteer population, brief treatment duration, and loading-dose strategy, which restrict conclusions regarding longer-term exposure and generalizability to patients receiving vortioxetine for major depressive disorder.

Mlyncekova et al. subsequently evaluated 30 adolescent inpatients with moderate-to-severe major depressive disorder using video-polysomnography before and after vortioxetine treatment [12]. Participants ranged from 12 to 18 years of age, with a mean age of 15.0 ± 1.5 years, and 21 were female. All had previously received fluoxetine, which was discontinued for a two-week drug-free period before baseline polysomnography. Although this interval reduced recent treatment exposure, residual fluoxetine or norfluoxetine effects may have persisted in some participants because of their prolonged elimination. Human sleep studies have shown that REM-related effects of fluoxetine can persist after discontinuation and normalize more slowly than some other sleep measures [19]. Residual exposure could therefore have influenced the baseline REM measures and, consequently, the magnitude of the subsequent baseline-to-treatment change; the direction of this potential effect cannot be determined from the available data. Vortioxetine was administered at 10 mg/day in 18 participants, 15 mg/day in 10 participants, and 20 mg/day in two participants, with repeat polysomnography performed after a median treatment duration of 21 days. REM latency increased from 132.0 ± 75.3 minutes at baseline to 193.0 ± 89.8 minutes after treatment (p = 0.005), while REM sleep percentage decreased from 20.10% ± 6.59% to 16.20% ± 6.33% (p = 0.009) [12]. In contrast, REM density and REM fragmentation were not significantly altered. No significant changes were identified in total sleep time, sleep efficiency, sleep latency, wake after sleep onset, arousal index, or the percentages of N1, N2, and N3 sleep, and the evaluated spectral measures likewise did not demonstrate significant treatment-related changes.

Depressive symptoms and insomnia severity also improved during the Mlyncekova et al. study, which increases the clinical relevance of the findings but complicates causal attribution [12]. In the absence of a placebo or untreated comparison group, changes between baseline and follow-up cannot be attributed exclusively to vortioxetine independent of improvement in depression-associated sleep abnormalities, potential residual effects of prior antidepressant exposure, or other time-related influences. The authors described the REM findings as apparently dose-related; however, the study was not designed as a randomized dose-ranging investigation, the dose groups were markedly unequal, and only two participants received 20 mg/day. The apparent dose-related pattern should therefore be regarded as hypothesis-generating rather than evidence of an established dose-response relationship. Additional limitations include the relatively small sample, adolescent inpatient population, and approximately three-week treatment duration.

Considered together, the two studies demonstrate directional convergence rather than direct replication. Both identified delayed entry into REM sleep together with reduced REM sleep expression during vortioxetine exposure, but the specific REM outcomes were not identical [11,12]. Wilson et al. demonstrated prolonged REM onset latency and reduced total REM duration under short-term placebo-controlled crossover conditions in healthy adults, whereas Mlyncekova et al. demonstrated prolonged REM latency and reduced REM percentage during several weeks of clinical treatment in adolescents with major depressive disorder. The Wilson et al. study provides stronger evidence for direct short-term pharmacodynamic attribution, while the Mlyncekova et al. study extends the observation to a clinically affected population but provides less certainty regarding whether the measured changes resulted from vortioxetine itself, improvement in the underlying disorder, or both. The absence of significant changes in REM density and REM fragmentation in the adolescent study further argues against interpreting vortioxetine as uniformly suppressing all dimensions of REM physiology.

Taken together, the available human evidence supports a vortioxetine-associated REM-related pharmacodynamic effect characterized principally by delayed REM onset and reduced REM sleep expression [11,12]. The current evidence does not establish uniform suppression across all REM-related measures, persistence of these alterations during long-term maintenance treatment, a clinically established dose-response relationship, or a receptor-specific mechanism responsible for the observed REM-related changes. It also does not establish that vortioxetine-associated REM changes represent the normalization of depression-related sleep abnormalities, mediate antidepressant efficacy, or independently predict subjective sleep quality, daytime functioning, cognitive outcomes, or other clinically meaningful benefits. A measurable pharmacodynamic change in REM architecture should therefore be distinguished from both a therapeutic mechanism and a clinical endpoint. The study designs, populations, vortioxetine exposures, principal polysomnographic findings, and interpretive limitations of the available human evidence are summarized in Table 3.

**Table 3:** Human polysomnographic studies evaluating vortioxetine and sleep architecture Quantitative values are presented as mean ± standard deviation where reported. The two studies demonstrate changes in selected REM-related measures but differ substantially in population, design, exposure, and outcome definitions and should not be regarded as direct replications. Absolute REM duration in the Wilson et al. study should be interpreted in relation to concurrent changes in TST, particularly under the 40-mg loading condition. REM latency, REM duration, REM percentage, REM density, and REM fragmentation represent distinct measures and should not be interpreted interchangeably. MDD: major depressive disorder; PSG: polysomnography; REM: rapid eye movement; TST: total sleep time; WASO: wake after sleep onset

Study	Population and design	Vortioxetine exposure	REM latency	REM quantity	Other PSG findings	Interpretation and limitations
Wilson et al. (2015) [11]	24 healthy young men; 19 completed all periods; randomized, double-blind, placebo-controlled, active-comparator, four-way crossover study	20 or 40 mg/day for 3 days as loading regimens intended to approximate different steady-state exposures; ≥3-week interval between periods	Night 3: 127 ± 45 minutes at 20 mg and 231 ± 65 minutes at 40 mg vs 63 ± 18 minutes with placebo; both p < 0.001 vs placebo	Total REM sleep: 63 ± 20 minutes at 20 mg and 41 ± 22 minutes at 40 mg vs 108 ± 21 minutes with placebo; both p < 0.001 vs placebo	TST: 403 ± 44 minutes at 20 mg and 393 ± 51 minutes at 40 mg vs 413 ± 29 minutes with placebo; TST was significantly reduced at 40 mg. Stage N1 increased; 40 mg also increased WASO	Supports a direct short-term pharmacodynamic effect on selected REM measures. Larger changes at 40 mg represent an exposure-related gradient rather than an established clinical dose-response relationship. Reduced TST at 40 mg may partly contribute to the lower absolute REM duration. Limited by small, male-only healthy-volunteer sample and brief loading-dose exposure
Mlyncekova et al. (2023) [12]	30 adolescent inpatients with moderate-to-severe MDD; uncontrolled before-and-after video-PSG study	10 mg/day (n = 18), 15 mg/day (n = 10), or 20 mg/day (n = 2); median treatment duration 21 days	Increased from 132.0 ± 75.3 minutes at baseline to 193.0 ± 89.8 minutes after treatment (p = 0.005)	REM percentage decreased from 20.10% ± 6.59% to 16.20% ± 6.33% (p = 0.009)	REM density: 3.12 ± 2.80 to 2.74 ± 2.29 (p = 0.931); REM fragmentation: 11.70 ± 7.07 to 12.50 ± 7.12 (p = 0.558). No significant changes in TST, sleep efficiency, N1, N2, or N3	Extends REM-related findings to a clinical population, but attribution is limited by the uncontrolled design, simultaneous clinical improvement, prior fluoxetine exposure, unequal dose groups, and short treatment duration. Residual fluoxetine/norfluoxetine effects may have influenced baseline PSG measures

Clinical implications

The principal clinical question raised by the available polysomnographic findings is not whether vortioxetine can alter REM sleep, but whether those alterations have independent therapeutic significance. Direct human evidence is limited to two substantially different studies: a short-term controlled study in healthy men and an uncontrolled before-and-after study in adolescents with major depressive disorder [11,12]. These findings therefore represent limited and heterogeneous evidence rather than replicated clinical confirmation across comparable populations and treatment conditions. Within that context, vortioxetine has been associated with measurable changes in REM latency and REM sleep expression [11,12]. However, a directionally convergent pharmacodynamic effect is not equivalent to a therapeutic biomarker. Across the broader antidepressant literature, REM-related effects vary among medications and pharmacologic classes and may occur alongside changes in other dimensions of sleep architecture [9,20-22]. A recent review similarly emphasized that serotonergic and other antidepressants can produce differing effects on REM sleep, sleep continuity, and overall sleep architecture, underscoring that an isolated REM alteration should not be assumed to represent a uniform or therapeutically meaningful sleep effect [22]. Current evidence therefore does not support interpreting the magnitude of vortioxetine-associated REM alteration as a surrogate for antidepressant response, remission, or long-term clinical outcome.

A second distinction concerns baseline REM physiology versus treatment-induced REM change. Major depressive disorder has been associated with abnormalities in REM latency, REM density, and the temporal distribution of REM sleep, but these measures represent different dimensions of REM regulation and should not be treated as interchangeable markers of depressive pathophysiology [7,20,22]. Baseline REM characteristics have also been investigated as potential predictors of treatment response. For example, greater pretreatment REM density has been associated with subsequent response to antidepressant pharmacotherapy [23]. Such findings concern the prognostic significance of a baseline physiologic characteristic, however, and do not establish that pharmacologically prolonging REM latency or reducing REM sleep mediates clinical improvement. Vortioxetine-associated REM changes therefore should not presently be interpreted as evidence of normalization of an underlying depression-related sleep abnormality.

Objective sleep physiology should likewise be distinguished from the patient's subjective sleep experience. In the adolescent clinical study, insomnia severity measured with the Athens Insomnia Scale decreased from 7.53 ± 5.73 before treatment to 4.70 ± 3.91 after treatment (p = 0.002), while the Children's Depression Inventory total score decreased from 20.9 ± 11.3 to 14.1 ± 10.0 (p < 0.001) [12]. These improvements occurred during the same treatment period in which REM latency increased and REM percentage decreased. However, because the study lacked a placebo or untreated comparison group, the parallel improvement in patient-reported symptoms and REM-related measures does not establish that the sleep-architecture changes mediated the clinical improvement. The healthy-volunteer study was designed primarily to characterize pharmacodynamic sleep effects rather than therapeutic or patient-important outcomes [11]. Accordingly, the available human evidence does not establish a quantitative relationship between the magnitude of vortioxetine-associated REM changes and subjective sleep quality, depressive improvement, daytime functioning, cognition, or treatment tolerability. Objective polysomnographic findings and patient-reported outcomes should therefore be considered complementary but distinct domains.

The differentiated pharmacology of vortioxetine also warrants cautious clinical interpretation. Wilson et al. demonstrated that vortioxetine and paroxetine differed in the relationship between estimated SERT occupancy and REM-related effects [11]. Together with the multimodal receptor pharmacology of vortioxetine and preclinical evidence involving 5-HT3 receptor antagonism, this observation provides a biologically plausible basis for pharmacodynamic differentiation from antidepressants acting predominantly through SERT inhibition [10,17,18]. However, pharmacodynamic differentiation does not establish therapeutic superiority. Available evidence does not demonstrate that vortioxetine's multimodal receptor profile confers clinically meaningful advantages in sleep quality, antidepressant efficacy, daytime functioning, cognition, or tolerability through its effects on REM sleep.

Accordingly, REM-related polysomnographic findings currently have no established role in routine clinical decision-making with vortioxetine. From a pharmacovigilance perspective, an objective change in REM architecture should not by itself be classified as an adverse drug reaction, a safety signal, or an evidence of therapeutic benefit. The two principal polysomnography studies did not establish that the observed REM-related changes were associated with insomnia, abnormal dreams or nightmares, somnolence, daytime impairment, treatment discontinuation, or other clinically meaningful safety outcomes [11,12]. In the adolescent study, depressive symptoms and patient-reported insomnia improved during treatment, but these parallel changes do not establish that the REM alterations produced the clinical improvement [12]. The healthy-volunteer study assessed short-term safety and tolerability but was not designed to determine whether REM changes predicted patient-important adverse effects or benefits [11]. There is therefore insufficient evidence to use REM latency, REM percentage, REM duration, or other REM measures to select vortioxetine over another antidepressant, determine an optimal dose, monitor treatment response, or predict clinical outcome. Treatment decisions should remain based on depressive symptom response, functional improvement, adverse effects, tolerability, and the patient's subjective sleep experience. Future investigations should determine whether serial changes in specific REM parameters correlate prospectively with antidepressant response, subjective sleep quality, daytime functioning, cognition, adverse effects, and longer-term outcomes. Until such relationships are demonstrated, vortioxetine-associated REM alterations are best regarded as objective pharmacodynamic changes whose independent clinical significance remains uncertain.

Limitations and future directions

Several limitations constrain the interpretation of the current evidence regarding vortioxetine-associated changes in REM sleep. Most importantly, direct human polysomnographic evidence is derived from two principal studies with substantially different populations and designs: a short-term controlled crossover study in healthy adult men and an uncontrolled clinical study in adolescents with major depressive disorder [11,12]. These investigations differ in age, clinical status, comparator conditions, vortioxetine exposure, treatment duration, and measured sleep outcomes, limiting direct comparison and making quantitative pooling inappropriate. The healthy-volunteer study provides stronger evidence for a direct short-term pharmacodynamic effect but has limited clinical generalizability, whereas the adolescent study provides greater clinical relevance but cannot fully distinguish medication-related effects from changes in depression-associated sleep abnormalities. In addition, baseline polysomnography in the adolescent study was performed only two weeks after fluoxetine discontinuation, and residual fluoxetine or norfluoxetine effects may have influenced baseline REM measures and therefore the apparent magnitude of change observed during vortioxetine treatment [12,19]. Neither study establishes the persistence of REM alterations during long-term maintenance treatment or their generalizability to broader adult populations with major depressive disorder.

The available studies also characterize only selected dimensions of REM physiology. Both assessed REM latency and measures of REM quantity, but the specific outcomes were not identical, and only the adolescent study evaluated REM density and REM fragmentation, neither of which changed significantly [11,12]. Delayed REM onset and reduced REM sleep expression therefore should not be generalized to uniform suppression of the REM state. Other aspects of REM physiology, including longitudinal adaptation, REM microstructure, night-to-night variability, and quantitative electrophysiologic measures, remain insufficiently characterized. Formal dose-response relationships are also unresolved. Although an exposure-related gradient was observed within the short-term Wilson et al. experiment and dose dependence was suggested in the adolescent study, the available evidence does not establish a clinically reproducible relationship between vortioxetine dose and specific REM outcomes [11,12].

Mechanistic interpretation remains similarly constrained. Human polysomnographic studies evaluate the integrated effects of vortioxetine as a complete drug and cannot isolate the contribution of SERT inhibition or individual 5-HT receptor actions. Pharmacologic and preclinical studies, particularly those involving 5-HT3 receptor antagonism, provide biological plausibility for mechanisms that may contribute to the observed REM-related pharmacodynamic changes [10,17,18]. However, these findings do not establish that 5-HT3 receptor antagonism, or any other individual pharmacologic action of vortioxetine, mediates the REM-related changes observed in humans. More broadly, the direct evidence base does not include controlled polysomnographic studies in typical adult patients with major depressive disorder receiving standard therapeutic doses of vortioxetine over clinically relevant treatment durations, limiting generalizability beyond the existing healthy-volunteer and adolescent clinical studies. The available evidence likewise does not establish whether vortioxetine-associated REM changes represent the normalization of depression-associated sleep abnormalities, mediate antidepressant response, or occur as a secondary pharmacodynamic consequence of treatment.

The clinical significance of the observed REM-related changes therefore remains uncertain. Existing studies have not established prospective relationships between the magnitude of REM change and antidepressant response, remission, subjective sleep quality, daytime functioning, cognitive performance, or quality of life [11,12]. They likewise do not establish prospective relationships between REM alterations and sleep-related adverse events, treatment-emergent insomnia, abnormal dreams or nightmares, somnolence, daytime impairment, or treatment discontinuation [11,12]. Accordingly, an objective polysomnographic alteration should not, by itself, be interpreted or classified as an adverse drug reaction in the absence of associated symptoms, functional impairment, or other evidence of clinical harm. Baseline REM characteristics investigated as potential predictors of antidepressant response should also be distinguished from treatment-induced REM alterations [23]. Determining whether vortioxetine-associated changes in REM physiology have independent clinical relevance will require studies designed specifically to relate serial objective sleep measures to validated clinical and patient-reported outcomes rather than inferring therapeutic significance from polysomnographic change alone.

Future investigations should prioritize adequately powered prospective studies using standardized polysomnographic acquisition and scoring methods, clearly defined treatment exposure, and serial assessments capable of distinguishing acute from persistent effects. Randomized placebo-controlled studies in adults with major depressive disorder would help separate direct medication effects from changes associated with improvement in depressive illness. Active-comparator studies using serotonergic antidepressants with differing pharmacologic profiles should additionally be designed to distinguish class-level effects on REM sleep from any vortioxetine-specific pharmacodynamic pattern under comparable treatment conditions, because a difference from placebo alone would not establish pharmacodynamic differentiation from other antidepressants. Formal dose-ranging designs are needed before dose-response relationships can be established. Future studies should assess REM latency, duration, percentage, density, fragmentation, and other relevant REM measures separately while integrating these outcomes with depressive symptoms, subjective sleep quality, daytime functioning, cognition, tolerability, and longer-term treatment outcomes.

Finally, this article is a focused narrative review rather than a systematic review or meta-analysis. Although the literature search and evidence-selection approach were structured to improve transparency, no formal risk-of-bias instrument was applied, and qualitative study selection and synthesis remain susceptible to selection bias. This limitation is particularly relevant to the mechanistic sections of the review, because the broader relevance-based selection of pharmacologic and preclinical literature may influence the apparent weight assigned to specific mechanistic hypotheses, including those involving 5-HT3 receptor antagonism. Accordingly, the prominence of a particular mechanistic explanation in the contextual literature should not be interpreted as evidence that it mediates vortioxetine-associated REM changes in humans. In addition, searches were restricted to English-language publications and relied on PubMed and Google Scholar, which may have introduced language and database-selection bias and may have resulted in the omission of relevant evidence indexed elsewhere. These methodological limitations, together with the small and heterogeneous direct evidence base, require that conclusions remain proportional to the available data. Until larger and methodologically comparable human studies are available, vortioxetine-associated REM alterations are best regarded as measurable pharmacodynamic effects whose persistence, dose dependence, mechanism, and independent clinical significance remain unresolved.

## Conclusions

The available human evidence indicates that vortioxetine is associated with measurable alterations in REM sleep architecture, but the evidence base remains limited and heterogeneous. These changes are best interpreted as a pharmacodynamic effect rather than evidence of a specific neurobiological mechanism or therapeutic effect. The available data are insufficient to determine the persistence, dose dependence, or broader generalizability of these alterations.

The clinical significance of vortioxetine-associated REM changes remains uncertain. Current evidence does not establish that these alterations mediate antidepressant efficacy, predict treatment response, or independently improve subjective sleep, daytime functioning, cognition, or other clinically meaningful outcomes. Larger prospective and controlled studies integrating standardized polysomnographic assessment with clinical and patient-reported outcomes are needed to determine whether these REM-related changes have independent clinical relevance or primarily reflect a secondary pharmacodynamic effect of treatment.
